# Optimizing treatment for diabetic macular edema during cataract surgery

**DOI:** 10.3389/fendo.2023.1106706

**Published:** 2023-01-25

**Authors:** Leo Ka Yu Chan, Sui Sum Lin, Fiona Chan, Danny Siu-Chun Ng

**Affiliations:** ^1^ Hong Kong Eye Hospital, Hong Kong, Hong Kong SAR, China; ^2^ Department of Ophthalmology and Visual Sciences, Faculty of Medicine, The Chinese University of Hong Kong, Hong Kong, Hong Kong SAR, China; ^3^ Department of Counselling and Psychology, Faculty of Social Sciences, Hong Kong Shue Yan University, Hong Kong, Hong Kong SAR, China; ^4^ Faculty of Medicine, The Chinese University of Hong Kong, Hong Kong, Hong Kong SAR, China

**Keywords:** diabetic macular edema (DME), cataract surgery, diabetic mellitus (DM), corticosteriods, anti VEGF

## Abstract

Diabetic macular edema (DME) causes visual impairment in diabetic retinopathy (DR). Diabetes mellitus is a global epidemic and diabetic individuals are at risk of developing DR. Approximately 1 in 10 diabetic patients suffers from DME, which is the commonest cause of vision-threatening DR at primary-care screening. Furthermore, diabetes predisposes to a higher frequency and a younger onset of cataract, which further threatens vision in DME patients. Although cataract extraction is an effective cure, vision may still deteriorate following cataract surgery due to DME progression or recurrence, of which the risks are significantly higher than for patients without concurrent or previous history of DME at the time of operation. The management of pre-existing DME with visually significant cataract is a clinical conundrum. Deferring cataract surgery until DME is adequately treated is not ideal because of prolonged visual impairment and maturation of cataract jeopardizing surgical safety and monitoring of DR. On the other hand, the progression or recurrence of DME following prompt cataract surgery is a profound disappointment for patients and ophthalmic surgeons who had high expectations for postoperative visual improvement. Prescription of perioperative anti-inflammatory eye drops is effective in lowering the risk of new-onset DME after cataract surgery. However, management of concurrent DME at the time of cataract surgery is much more challenging because DME is unlikely to resolve spontaneously even with the aid of anti-inflammatory non-steroidal or steroid eye drops. A number of clinical trials using intravitreal injection of corticosteroids and anti-vascular endothelial growth factor (anti-VEGF) as first-line therapy have demonstrated safety and efficacy to treat DME. These drugs have also been administered perioperatively for the prevention of DME worsening in patients undergoing cataract surgery. This article reviews the scientific evidence to guide ophthalmologists on the efficacy and safety of various therapies for managing patients with DME who are particularly vulnerable to cataract surgery-induced inflammation, which disintegrates the blood–retinal barrier and egression of fluid in macular edema.

## Introduction

### Epidemiology of diabetes mellitus

Diabetes mellitus (DM) is a chronic disease common worldwide, characterized by hyperglycemia due to impaired glucose regulation. People with type 1 diabetes mellitus (T1DM) are unable to produce sufficient insulin, whereas people with type 2 diabetes mellitus (T2DM) suffer from end-tissue resistance to the effects of insulin (1). DM is a serious public health issue that continues to place a high burden on patients and healthcare systems, thanks to a constant rise in its prevalence.

According to the International Diabetes Federation (IDF), the total number of people having DM (T1DM and T2DM combined) rose constantly from approximately 285 million people in 2009 to 366 million in 2011, 382 million in 2013, 415 million in 2015, and 425 million in 2017 (2–6). In 2019, 463 million people were estimated to live with DM globally, which accounted for 9.3% of the global adult population (20–79 years). Moreover, this number is expected to spring to 578 million (10.2%) in 2030 and 700 million (10.9%) in 2045 (7).

Research reported regional differences among the DM population. In terms of prevalence, Pacific Ocean Island nations maintained first place (8). For instance, Fiji, Mauritius, American Samoa, and Kiribati had prevalence rates of 20,277, 18,545, 18,312, and 17,432 per 100,000, respectively. In terms of the greatest total number of individuals with DM, China, India, and the US remained the top countries with 88.5 million, 65.9 million, and 28.9 million individuals with T2DM, respectively, due to their large population size. In terms of the greatest increase, the WHO reported that low- and middle-income countries, like Indonesia, Malaysia, Thailand, and Vietnam, had maintained their ranking in the last two decades.

### Epidemiology of diabetic retinopathy

The inability to regulate blood sugar levels damages different body parts and leads to a multitude of complications, including but not limited to cardiovascular disease, neuropathy, and retinopathy. Here, we will first focus on how DM induces diabetic retinopathy (DR), as the eye is the organ where DM potentially first manifests and, hence, is a reflection of systemic diseases.

DR is recognized as the leading cause of vision loss in the working-age population in both developed and developing countries (9). DR is characterized by vascular abnormalities in the retina and is classified into two stages: non-proliferative diabetic retinopathy (NPDR) and proliferative diabetic retinopathy (PDR). NPDR and PDR are also identified as vision-threatening diabetic retinopathy (VTDR).

Among the approximately 463 million DM population, approximately one-third exhibited signs of DR (10). The literature reported that up to 2020, the global prevalence of DR was 22.27%, among which 6.17% of patients are susceptible to vision loss from VTDR and 4.07% from clinically significant macular edema (CSME) (11). The global numbers of DR, VTDR, and CSME are expected to further escalate to 160.50 million, 44.82 million, and 28.61 million, respectively, by 2045. Africa had the highest rate of DR (35.90%), followed by North America and the Caribbean (33.30%), and finally South and Central America with the lowest rate (13.37%) (10). Hispanics and Middle Easterners who are diabetic showed higher susceptibility toward DR than Asians. In this regard, an Italian study group showed that among 745 diabetic patients undergoing phacoemulsification, NPDR, PDR, and laser-treated retinopathy were present in 101 (14.3%), 13 (1.7%), and 53 (7.5%) patients, respectively (12). Furthermore, there was a positive correlation between the duration of DM and the severity of DR (13).

### Epidemiology of diabetic macular edema

Diabetic macular edema (DME) is defined by the breakdown of the blood–retinal barrier (BRB) causing swelling or thickening of the macula due to sub- and intraretinal accumulation of fluid (14). DME is the primary cause of vision loss in patients with DR (9). Elevated HbA1c is known to be a significant risk factor for diabetic retinopathy (15). Hence, the control of HbA1c levels is critical in DME. Among the one-third of the DM population who demonstrated signs of DR, a further one-third of them experienced VTDR, including DME (10). As there is a rising number of diabetes, DME is anticipated to pose a major threat to the public health system in the foreseeable future.

With the aid of various diagnostic modalities, such as slit lamp biomicroscopy, fundus photography, and optical coherence tomography (OCT), immense effort has been made to quantify DME. In particular, OCT outstands other tools with its supreme accuracy of measurement of retinal thickness and high resolution for monitoring of retinal changes on a microscopic level (16, 17). Therefore, OCT was considered as the gold standard for the diagnosis and prognosis monitoring of DME (18). While the prevalence of DME varied greatly among studies due to different diagnostic tools and criteria used, Im et al. focused on OCT-diagnosed DME and only included population-based studies to avoid skewed prevalence from hospital- and/or clinical-based samples (19). In that study, among diabetic patients, Im et al. proposed the overall pooled prevalence of DME was 5.47%, 5.81% for low-to-middle-income countries, and 5.14% for high-income countries. In contrast to DM or DR, the statistical difference in the prevalence of DME between high-income and low-to-middle-income countries was insignificant.

### Epidemiology of cataract in DM patients

Cataract is the clouding of the crystalline lens and can be further differentiated according to types, such as nuclear, cortical, and posterior subcapsular cataract (20). The incidence of cataract formation was proved to be inflated among diabetic patients (21). With the advent of technological advancement, cataract surgery has gradually become a much safer procedure over the centuries to improve patients’ vision. Despite this, postoperative complications are still inevitable and may lead to unsatisfactory visual outcomes. Examples include postoperative DME, DR progression, and posterior capsular opacification (22).

Research showed that diabetic patients are two to five times more prone to earlier onsets of cataract when compared with the control group (23–26). In a study conducted based on the UK population, the incidence rates of cataract were 20.4 per 1,000 person-years (py) for the diabetic population, which almost doubled that of the general population, of which the baseline was 10.8 per 1,000 py (27). Furthermore, the incidence rate ratio peaked for patients 45 to 54 years old. Moreover, the longer the duration of having DM, the higher the risk of developing cataract.

Consistently, a community-based cross-sectional study done in Saudi Arabia showed that among 668 eyes from 334 patients with T2DM, 237 eyes (35.5%) had cataract (28). Similar to the findings of Becker et al., diabetic patients with cataract were associated with a longer duration of diabetes. Furthermore, DR was found in 215 diabetic cataract eyes (32.2%). Among them, 194 eyes (90.2%) were NPDR and 89 eyes (13.3%) were CSME.

## Association of DME and cataract surgery

### Pathophysiology (breakdown of the blood–retinal barrier)

Although the exact mechanism of the action of DR remains ambiguous, a considerable amount of prospective clinical studies have proved that hyperglycemia is the primary risk factor contributing to the pathogenesis of DME (29). Four major biochemical pathways were identified to be related to the hyperglycemia-induced pathogenesis of DR: 1) polyol pathway, 2) advanced glycation end products pathway, 3) protein kinase C (PKC) pathway, and 4) hexosamine pathway (30). These four pathways trigger heightened oxidative stress, inflammation, and vascular dysfunction. Oxidative stress and inflammation induce hypermodulation of growth factors and cytokines, which contribute to the breakdown of the BRB and the formation of DME. For instance, vascular endothelial growth factor (VEGF), angiopoietins, tumor necrosis factor (TNF), interleukins (ILs), and matrix metalloproteinases (MMPs) are the key modulators. The BRB plays a prominent role in maintaining the fluid electrolyte equilibrium in the retina. However, when the BRB is broken down, fluid accumulates in the different layers of the retina, leading to DME. Anatomically, the BRB is divided into outer and inner layers. The outer BRB is formed by retinal pigment epithelium (RPE) cells between the fenestrated choriocapillaris and the outer retina, whereas the inner BRB is composed of endothelial cells situated at the inner retinal capillaries. At the outer BRB, the RPE has been shown to eliminate water from the subretinal space toward the choroid *via* a mechanism driven by an active trans-epithelial Cl– gradient (31). At the inner BRB, the tight endothelial cell–cell junctions avoid molecular leakage from the retinal capillaries and, thus, play a critical role in the retinal hydro-ionic homeostasis. The cohesion of the cell–cell junctions is dynamically maintained by an intricate neuro-glio-vascular cross-talk between retinal Müller glial (RMG) cells and astrocytes, and their interactions with the surrounding smooth muscle cells and pericytes (32–34). With various ion and aqueous channels, the RMG cells contribute significantly to the regulation of fluid homeostasis (35). Together, an imbalance between fluid entry secondary to the breakdown of the BRB and dysfunctional fluid withdrawal of the RPE and RMG results in an upset fluid electrolyte equilibrium with a net gain of fluid and, hence, DME (36). In DME, the breakdown of the cell–cell junctions, pericyte loss, and thickening of the basement membrane are observed (37).

### Incidence of new-onset DME after cataract surgery

As mentioned above, diabetic patients are more liable to develop cataracts. Research has shown that cataract surgery improves best-corrected visual acuity (BCVA) and vision-related quality of life in patients with DR (38). Meanwhile, patients with DR are more predisposed to poorer postoperative visual acuity and a higher risk of complications after cataract surgery when compared with those without DR (39–42). This is substantiated by a large database study of 81,984 eyes done in the UK that showed that there was an increased incidence of new-onset DME after cataract surgery (39). Among 4,485 diabetic eyes in the absence of preoperative maculopathy that underwent cataract surgery within 90 days, 2,807 (62.6%) of them did not have DR after surgery, while 1,678 (37.4%) of them suffered from postoperative DR. The data showed that diabetic patients, even with no retinopathy, had a higher relative risk of new DME onset of 1.80 after cataract surgery when compared with the control. The risk was even higher (6.23) in the presence of any pre-existing DR. The risk of developing postoperative DME is directly proportional to the severity of DR. Furthermore, the mean incidence of postoperative edema in the eyes of diabetic patients was found to be fourfold in comparison with non-diabetic patients (39).

### Incidence of recurrent DME after cataract surgery and pre-existing DME progression after cataract surgery

A large cohort study done in Italy recruited a total of 3,657 patients who underwent cataract surgery in the past 3 months (12). Among the cohort, 745 (20.4%) patients were diabetic. Men had a significantly higher prevalence of DM (24.7%) than women (17%). Within the 745 diabetic patients, 205 (27.5%) patients showed signs of DME, among which 156 (20.9%) patients had non-clinically significant macular edema (N-CSME) and 49 (6.6%) patients had CSME. N-CSME was defined as the presence of intraretinal cysts associated with the center foveal thickness (CFT) of 257 µm, which was equivalent to 30% thicker than normal values. CSME was defined by the presence of intraretinal cysts associated with CFT of 598 µm, which was equivalent to >30% thicker than normal values. Patients with DME had a significantly longer history of DM, but no significant difference between gender or age groups was identified (13). More importantly, among the 3,657 patients, the prevalence of DME was 5.4%. Although this was not a population-based study, the prevalence of DME was consistent with the proposed general prevalence of DME of 5.4% in Im et al. as stated previously.

Apart from the incidence of DME after cataract surgery, it is also essential to understand how DME progresses, which is reflected by visual acuity after cataract surgery in patients with different degrees of DR. Research evaluated diabetic patients’ change in BCVA throughout a year after cataract surgery (43). Diabetic eyes without DR before surgery (*n* = 138) and eyes with NPDR (*n* = 125) gained a median of 11.0 and 10.0 Early Treatment Diabetic Retinopathy Study (ETDRS) letters from 65.0, respectively. Eyes with severe NPDR (*n* = 20) and PDR (*n* = 72) gained 20.5 and 15.0 letters from 55.0, respectively. Compared with eyes with severe NPDR or PDR, eyes without DR or mild/moderate NPDR had significantly greater improvements in VA when controlling for baseline VA. As a result, patients with a more severe degree of DR might result in poorer visual acuity even after cataract surgery. The conundrum of whether to offer cataract to diabetic patients remained controversial.

## Management of DME and cataract surgery

In diabetic patients who underwent cataract surgery, macular edema can be resulted either from a new onset of pseudophakic cystoid macular edema (PCME) or the worsening of pre-existing DME. Both entities are characterized by fluid accumulation in the retinal tissues in the macular region, but these two diseases should be distinguished as they have different pathophysiologies and, hence, different treatment paradigms. DME often presents with an underlying DR, exudates, and macular edema (ME), while minimal DR and the absence of exudates point more toward PCME (44). To further differentiate between the two, OCT is an invaluable diagnostic tool. For DME, OCT shows such features as microaneurysms, hard exudates, and a higher parafoveal outer nuclear layer to inner nuclear layer thickness ratio, whereas for PCME, OCT demonstrates a high central macular thickness to retinal volume ratio and intact hyperreflective outer retinal bands (45).

As the pathophysiologies of DME and PCME are different, the treatments for DME and PCME differ. In this context, PCME is mostly managed with topical treatments, whereas DME is managed with more invasive treatments such as intravitreal injections and laser photocoagulation. Boscia et al. suggested that all diabetic patients undergoing cataract surgery should be treated with topical non-steroidal anti-inflammatory agents (NSAIDs) to prevent PCME. As for patients with pre-existing DME, intravitreal therapies, both with anti-VEGF drugs and steroids, can be considered (46). The perioperative treatment options for DME in patients with cataract have been summarized in **Table 1**.

**Table 1:** Perioperative treatment options for DME in cataract patients.

Treatment options	Clinical pearls and recommendations	References
Topical non-steroidal anti-inflammatory drugs (NSAID)	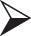 Agents: nepafenac, diclofenac, bromfenac, and ketorolac 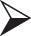 Perioperative use is recommended in eyes without preoperative DME to reduce the risk of developing DME postoperatively	(45, 47–49)
Topical corticosteroids	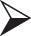 Lower penetration to the eye compared with NSAID 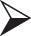 Combined use of topical corticosteroid and NSAID was superior to either agent alone	(52–54)
Laser	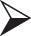 Lasers: focal, grid, subthreshold micropulse 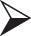 Considered as an adjunct treatment for refractory DME	(55–58)
Intravitreal corticosteroids	Triamcinolone acetonide (TA) 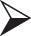 Demonstrated longer duration of action than intravitreal bevacizumab for the control of DME 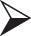 Preoperative use may hasten cataract progression 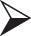 TA has a higher risk of increasing IOP	(62, 63)
	Fluocinolone acetonide (FA) implant 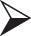 The benefit of FA has been demonstrated in clinical trials 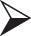 Recommended for use in pseudophakic and chronic DME patients refractory to other therapies Intravitreal dexamethasone implant (Ozurdex) 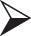 Intraoperative use is effective in the prevention of post-cataract surgery macular edema, with the effect lasting for up to 3 months 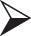 Preoperative use also improved post-cataract surgery visual acuity significantly	(76–78) (83, 84)
Subtenon TA	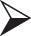 Decreased CMT significantly for the prevention of postoperative progression of DME 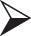 A viable treatment option in cases of DME refractory to intravitreal anti-VEGF	(73, 74)
Intravitreal anti-VEGF	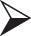 First-line treatment to control preoperative DME 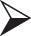 Treatment still needs to be continued following surgery for the control of DME	(64, 65, 68, 69)

### NSAID eye drops

Given the incidence of new-onset DME after cataract surgery, the perioperative use of anti-inflammatory eye drops is recommended. Topical NSAIDs block cyclooxygenase enzymes, which in turn hinder prostaglandin production. This reduces vascular hyperpermeability and, hence, decreases the incidence and severity of macular edema.

Currently, the common options of NSAID eye drops include nepafenac, diclofenac, bromfenac, and ketorolac. Nepafenac is a prodrug that penetrates the cornea rapidly and forms the active metabolite, amfenac. Out of these four agents, nepafenac displays higher permeability, greater duration of action, and increased targeted activation. Topical nepafenac can be given in 0.1% formula three times a day or in 0.3% formula once a day. Both formulations have been proven to be effective against PCME development. In a randomized, double-masked study involving 263 adult patients, a significantly lower percentage of patients on 0.1% nepafenac developed ME compared with the vehicle group over 90 days (3.2% and 16.7%, respectively, *p* < 0.001). The central macular thickness (CMT) increase and the change of macular volume from baseline were also significantly better in the nepafenac group over 14 days (*p* < 0.005) (47). Similar results were found in patients using 0.3% nepafenac, with the incidence of developing ME in the treatment and control groups being 4.1% and 15.9%, respectively (*p* < 0.001). No unanticipated safety events occurring in both trials were observed (48). Nepafenac has been approved in Europe and the Americas for the reduction of PCME development in diabetic patients (45).

The clinical benefits were also evident in the other types of NSAIDs as well. Alnagdy et al. stated that among diabetic patients undergoing cataract surgery, patients on topical NSAIDs, either ketorolac tromethamine 0.4% or nepafenac 0.1%, showed statistically significant improvement in BCVA (*p* = 0.04) and CMT over 3 months (*p* = 0.004) as compared with control without NSAIDs. There was no statistical difference in the efficacy between ketorolac and nepafenac (49). A retrospective analysis of 75 diabetics was also performed to investigate the effect of 0.1% bromfenac sodium hydrate. When compared with the control group over 6 months, bromfenac had better best-corrected visual acuity (0.12 ± 0.12 *vs*. 0.32 ± 0.42, *p* = 0.142), lower macular volume (8.46 ± 0.60 *vs*. 9.14 ± 1.53 mm^3^, *p* = 0.022), and lower central macular thickness (265.58 ± 31.28 *vs*. 314.15 ± 76.11 μm, *p <*0.001) (50).

NSAIDs are associated with side effects such as transient burning sensation and epithelial corneal defects (51). However, this side effect profile is relatively insignificant when compared with other treatment options, as there is no risk of endophthalmitis as in intravitreal injection and no risk of destruction of the foveal center as in laser surgery.

### Topical corticosteroids

Corticosteroids suppress inflammation by inhibiting COX-2 and phospholipase A2 and, hence, lipoxygenase pathways. A study conducted in Croatia involving 55 patients has demonstrated that topical diclofenac effectively lowered intraocular IL-12 concentration, a marker for intra-ocular inflammation, and reduced ME formation (52).

Although the mechanism of action of topical steroids is similar to those of NSAIDs, a topical steroid is more inferior in the prevention of PCME, probably due to its lower penetration in the eye. Moreover, steroids exhibit more severe side effects when compared with NSAIDs, such as increased intra-ocular pressure (IOP). Hence, the prolonged use of topical steroids should be avoided.

Despite its inferior effect when used alone, steroid eye drops can be used in combination with other treatments. A meta-analysis involving seven trials showed that in diabetic patients with no pre-existing DME, combining topical NSAIDs with corticosteroids reduced the risk of developing PCME to a greater extent versus tropical corticosteroids alone (OR = 0.17) (53). Similar improvements were observed when topical NSAIDs and steroids, bromfenac and dexamethasone, were used in combination. A multicenter trial involving 12 European centers compared the incidence of developing PCME over 12 weeks postoperatively in patients treated with bromfenac, dexamethasone, or in combination. The incidence was 3.6%, 5.1%, and 1.5%, respectively (overall *p* = 0.043). Bromfenac had a lower incidence of PCME development than dexamethasone, and the combined treatment had the lowest incidence overall (54).

### Laser

For patients with pre-existing DME, the treatment of DME is recommended preoperatively to reduce the risk of further progression.

The first prospective randomized clinical trial on laser photocoagulation—Early Treatment Diabetic Retinopathy Study (EDTRS)—examined 37,111 patients across 22 centers. It classified laser treatment into two techniques: focal and grid laser (55). Focal laser involved the treatment of focal lesions, such as microaneurysm, intraretinal microvascular abnormalities, and short capillary segment fluorescein leakage. Focal laser utilizes moderate intensity burns of 50 to 100 μm lasting 0.05 to 0.1 s in duration. Grid lasers are usually placed in the papillomacular bundle rather than the macular center or disc margin. The laser is of mild intensity with a sport size of 50 to 200 μm, lasting 0.05 to 0.5 s. There is also a modified ETDRS treatment approach, which uses a less intense laser with greater spacing.

The ETDRS concluded for clinically significant DME that focal photocoagulation should be considered (56). It defined clinically significant macular edema as retinal thickening at or within 500 microns from the macular center, or hard exudates at or within 500 microns of the macular center with adjacent retinal thickening, or retinal thickening greater than 1 disc diameter and within 1 disc diameter away from the macular center.

Mild macular laser photocoagulation (MMG) is a new modality of laser photocoagulation. Two hundred to 300 burns are applied to the entire area over the macular, both thickened and unthickened retina, and microaneurysms are directly photocoagulated. However, there was no evidence suggesting that MMG has a better outcome in terms of visual acuity or retinal thickening on follow-up after 12 months (57). Subthreshold diode micropulse laser photocoagulation is another technique to treat DME, with the aim to reduce laser damage to ocular tissues. The laser parameters are modified, such as decreased wavelength, retinal irradiance, and pulse duration, to reduce chorioretinal damage. The laser energy is given in pulses, lasting 300 ms each. In a study by Ulbig et al., 82% of patients treated with diode laser had completely or partially resolved DME (58). However, most trials on micropulse subthreshold diode therapy are non-randomized, uncontrolled, and retrospective and, hence, are of insufficient power for application in clinical practice. This relatively novel treatment modality still warrants further studies before its application in clinical settings.

There are also general complications of laser treatments which need to be considered. An important complication is the enlargement of a laser scar, which can threaten visual acuity. Maeshima et al. also reported that the expansion of laser scars was relentless and might continue over long time periods. The expansion rate was 8.8% during the first 4 years but then thereafter increased to 16.5% (59). Other complications include a transient increase of DME, accidental foveal burns, or choroidal neovascularization due to damage to Bruch’s membrane (57).

Prior to the era of intravitreal injections, laser treatments were considered as the gold standard that improved long-term visual acuity outcomes for most patients. Although anti-VEGF shows better resolution of DME after the first year, in the EDTRS study, the best results were achieved on follow-up after 3 years (60). Therefore, laser treatments still play a role in the treatment of DME during cataract surgery, especially in the long term.

### Triamcinolone acetonide versus anti-VEGF

Triamcinolone acetonide (TA) is a commonly used corticosteroid for intravitreal injections. The mechanism of TA is postulated to inhibit both inflammatory and angiogenic cytokines, hence an improvement in BCVA and a reduction in CMT.

When compared with anti-VEGF, TA has been shown to be more inferior. This is mostly due to the concerns raised by the side effect profile of TA. Intravitreal steroids pose a risk of transient increased IOP and endophthalmitis, with the prevalence of increased IOP up to 23.5% (60). In another study involving 12 patients, four patients showed increased IOP at 1 month after surgery. However, most of the IOP spike was manageable, as the IOP returned to normal without medication 6 months after the application of topical anti-glaucomatous drugs (61).

In comparison with anti-VEGF, intravitreal steroids may have a longer duration of action and possibly better control of macular thickness. In a prospective pilot study involving 41 DME patients, the visual outcomes between intravitreal bevacizumab (BVB) and TA administered intraoperatively were compared (62). After 6 months, there was no significant difference between the groups in terms of vision improvement. In the TA group, 69.9% of the patients were able to achieve visual acuity improvement of 15 letters or more at 6 months, as compared with 60.0% in the BVB group (*p* = 0.728). For a 10-letter improvement, the numbers were 82.6% and 73.3%, respectively (*p* = 0.687). However, only TA showed a sustained reduction in CMT. Three patients (12.5%) in the TA group experienced increased IOP compared with none in the BVB group. However, 70.6% of the participants in the BVB group required additional injections, compared with 16.7% in the TA group, suggesting that TA has less injection need in the long run.

This result was also supported by another randomized trial by Kandasamy et al. When TA and BVB were given in cataract surgery, both TA and BVB showed improved BCVA. TA and BVB patients had a letter gain of 21.4 and 17.3, respectively. However, only TA has sustained improvement in CMT, with only 24% of the patients requiring retreatment, when compared with 57% in the BVB group (63).

Other anti-VEGF agents were also investigated. Ranibizumab has been shown to be more effective when injected intraoperatively during cataract surgery than perioperatively and postoperatively in patients with DMR (64). Intraoperative aflibercept did not exert a significant effect on postoperative CMT or visual acuity at 3 months, probably due to a relatively shorter half-life (65). To date, there are no clinical trials yet examining the role of intraoperative injection of newer anti-VEGF agents, such as brolucizumab and faricimab on DMR, but their safety profiles and efficacies on DME were demonstrated in clinical trials (66, 67). Nevertheless, intravitreal anti-VEGF still remains the well-established first-line treatment for preoperative DME (68, 69). Further anti-VEGF treatment following cataract surgery still needs to be continued for the control of DME.

Despite anti-VEGF being more effective, not all patients demonstrate a response to anti-VEGF treatments. In a subanalysis of the DRCR.net Protocol I study, approximately 20% of patients had less than 20% reduction in CMT over a 1-year period. The study defined this as non-responders of ranibizumab therapy (70). Nunome et al. investigated the role of TA in DME treatment in ranibizumab non-responders (71). There was a significant improvement in visual acuity at 24 weeks, central retinal thickness (CRT) at 12 weeks, and retinal sensitivity threshold at 4 weeks in ranibizumab non-responders (71). This illustrates that TA combined with cataract surgery is useful for patients with anti-VEGF resistance.

Of note, TA can also be used in conjunction with other treatment modalities such as macular laser. Ozgur et al. reported that patients treated with IVTA and macular grid photocoagulation had a statistically significant increase in BCVA and a decrease in CMT at 6 months of follow-up, when compared with those who received macular laser alone (*p* < 0.01) (72). Furthermore, subtenon TA has been shown to decrease CMT significantly for the prevention of postoperative progression of DME (73). In this regard, subtenon TA is a viable treatment option in cases of DME refractory to intravitreal anti-VEGF (74).

### Fluocinolone acetonide

Another corticosteroid alternative is intravitreal fluocinolone acetonide (FA). The Fluocinolone Acetonide in Diabetic Macular Edema (FAME) study is a landmark trial for FA. The trial demonstrated that after intravitreal injection of an FA implant which releases 0.2 μg FA per day, 34% of patients with DME over 3 years experienced a >15 letter gain compared with 13.4% in the sham group. There was a 140-μm reduction in CRT after 6 months of treatment (75).

Another study comparing the long-term benefits of high-dose versus low-dose FA also concluded that FA improved BCVA in patients with DME over 2 years. The mean improvement in BCVA score from baseline in the low-dose, high-dose, and sham groups was 4.4, 5.4, and 1.7, respectively (*p* = 0.02 and *p* = 0.016 compared with sham). The study concluded that FA could be administered to patients with benefits lasting for at least 2 years (76).

It should be noted that intravitreal corticosteroids favor cataract formation. Both trials failed to take into account the cataract status of the patients. Currently, the National Institute for Health and Care Excellence recommends that FA should be used in pseudophakic patients and chronic DME refractory to other therapies. In the US, FA is approved for treating refractory DME, provided that patients have been treated with a course of corticosteroids without clinically significant IOP rise (77).

In the context of IOP rise, studies suggested that the prevalence of IOP elevation was higher in FA (65.9%–79.0%) compared with TA (30.0%–45.9%) (71). Despite this, the findings from a *post-hoc* analysis of the FAME study supported the use of FA implants in both phakic and pseudophakic patients. For phakic patients with DME, cataracts developed at an expectedly high rate, and surgery was needed. However, the results suggested that the visual outcomes were not negatively affected by the cataract surgery. There was numerically a higher increase in BCVA scores and >15 letter improvement compared with those who were pseudophakic at baseline. Although more research is needed, the analysis suggests that FA may protect the patient against post-cataract surgical complications and is favorable for long-term visual outcomes (78).

### Intravitreal dexamethasone implant (Ozurdex)

Ozurdex (Allergan, Inc., Irvine, CA, USA) is a single-use, biodegradable intravitreal dexamethasone drug-release system that releases a total dose of 700 μg of dexamethasone to the human vitreous slowly and gradually over time (79–82). Composed of a biodegradable copolymer with polylactic-co-glycolic acid and micronized dexamethasone, Ozurdex was engineered to overcome drug delivery barriers by lengthening the effect of intravitreal dexamethasone. A study examining the pharmacokinetics of Ozurdex in monkey eyes demonstrated that the intravitreal concentrations of Ozurdex were characterized by two distinct phases, with peak concentration attained at day 60 and subsequent continuous release up to day 180 (79). As Ozurdex is administered intravitreally, the possible side effects brought upon by steroid administration *via* other routes of administration, such as systemic administration, could be reduced. Furthermore, the biodegradability of the implant eliminates the need for the removal of the implant, as the implant gradually degrades into water and carbon dioxide.

### The effect of Ozurdex implant on diabetic macular edema after cataract surgery

For eyes with at least mild diabetic retinopathy and the absence of macular edema, an immediate intraoperative single Ozurdex injection after phacoemulsification was demonstrated to be effective in the prevention of macular edema by reducing the likelihood of CRT rise (83). Such an effect was observed to last for up to 3 months post-treatment, as evidenced by central retinal thickness, macular volume measurements with OCT, and improvement in best-corrected visual acuity (83). Furthermore, statistically significant improvement in visual acuity in groups of diabetic patients who received Ozurdex injection before phacoemulsification was also observed at 6, 12, and 24 weeks in comparison with the control (84). Meanwhile, there were no significant differences in intraocular pressure between the two groups.

The majority of adverse events associated with intravitreal dexamethasone implant injection are related to the injection per se and often resolve spontaneously (85). The common adverse effects include post-injection conjunctival hemorrhage, hyperemia, and chemosis, as well as raised intraocular pressure, and less commonly iritis, anterior chamber cell, and vitreous hemorrhage. The migration of the Ozurdex implant to the anterior chamber is a severe but rare complication. This could lead to corneal endothelial damage, corneal edema, and permanent decompensation, in which case corneal transplantation might be warranted. Immediate removal or repositioning of the implant should be performed urgently to avoid irreversible corneal endothelial damage. A study involving 640 eyes which received intravitreal dexamethasone implant injections revealed that anterior chamber implant migrations occurred in four eyes (0.63%) (86). The study identified the major risk factors for anterior chamber migration to be insufficient zonular support, defects or a non-intact posterior capsular membrane, and a history of vitrectomy. For patients with these risk factors, alternative treatments should be offered. Overall, Ozurdex was generally considered to be well-tolerated with a good safety profile (81, 85).

### The mechanism of corticosteroids

The exact mechanism of how pseudophakic cystoid macular edema occurs still remains unclear. The literature suggested that such inflammatory mediators as VEGF could potentially play a pivotal role in breaking down the blood–aqueous and blood–retinal barriers, thus resulting in increased vascular permeability and cystoid macular edema (87). In this regard, intravitreal corticosteroid alleviates diabetic macular edema by targeting the inflammatory cascade *via* diminishing the production and release of VEGF and other pro-inflammatory mediators, thereby hindering the formation of diabetic macular edema among diabetic patients who received cataract surgeries.

## Discussion

Cataract surgery helps patients restore their vision and improves their quality of life. The increased risk of postoperative macular edema in diabetic patients, especially in the presence of pre-existing DME, often leads to suboptimal vision gain and patient dissatisfaction. The perioperative control of the systemic cardiovascular risk factors, such as diabetes, blood pressure, and lipids, is critical to reduce the risk of postoperative DME and postoperative endophthalmitis, as well as to promote corneal wound healing and hasten vision recovery. Intraoperative factors including a non-intact posterior capsule, prolapse or incarceration of vitreous causing macular traction, iris chafing secondary to a malpositioned intraocular lens, retained lens matter, and prolonged operation time with extensive surgical manipulations should also be noted, as these may increase the risk of postoperative macular edema.

The options of prophylaxis for postoperative macular edema include topical NSAID, topical/periorbital/intravitreal steroids, or intravitreal anti-VEGF injections. For diabetic patients without a history of DME, the preoperative use of topical NSAID for 1 week reduces the risk of new-onset DME during the early postoperative period. The addition of a topical steroid did not have a significant effect in lowering the chance of postoperative DME but should be prescribed to suppress other forms of intraocular inflammation during the postoperative period.

For patients with pre-existing DME, if the cataract is not jeopardizing the patients’ activity of daily living (ADL) and there is an adequate fundal view, it is preferable to defer cataract surgery and control DME first, by achieving a static central foveal thickness on OCT on two consecutive monthly visits. Because of the short half-life of intravitreal anti-VEGF, injection within 14 days before cataract surgery is most efficacious in reducing macular thickness during the first postoperative month. Subtenon injection of triamcinolone acetonide has a longer half-life and should be given earlier. If the cataract is visually debilitating, affecting the ADL, or precludes fundal examination, then prompt cataract surgery is recommended with intravitreal anti-VEGF injection or intravitreal/subtenon injection of steroids. Of note, intravitreal dexamethasone implant has the risk of migrating into the anterior chamber if the posterior capsule is non-intact.

In the postoperative period, macular edema is the end result contributed by a combination of factors including postoperative inflammation and diabetes and often requires additional treatments, such as intravitreal or periocular steroids and intravitreal anti-VEGF injections. Patients who received anti-VEGF injections before cataract surgery can still experience improvements in vision postoperatively and can continue to receive anti-VEGF injections in the perioperative period.

## Author contributions

All authors wrote sections of the manuscript. All authors contributed to the manuscript revision and read and approved the submitted version.
